# Loneliness and time abroad in Polish migrants in the UK: Protective role of religious experience

**DOI:** 10.1371/journal.pone.0279984

**Published:** 2023-02-15

**Authors:** Małgorzata Tatala, Marcin Wojtasiński, Konrad Janowski

**Affiliations:** 1 Institute of Psychology, The John Paul II Catholic University of Lublin, Lublin, Poland; 2 School of Human Sciences, University of Economics and Human Sciences in Warsaw, Warsaw, Poland; University of Exeter, UNITED KINGDOM

## Abstract

The purpose of this study was to examine the relationship between time spent abroad, level of religious experience, and loneliness in Polish migrants in the UK. Factors differentiating the level of loneliness were migration time (up to one year, from one to five years, and over five years) and religious experience, which was postulated to have a protective function against the level of loneliness experienced. The R-UCLA test was used to verify the level of loneliness, and religious experience was measured with the Religious Experience Scale in participants (N = 200) who were Polish migrants. The results showed that the relationship between time abroad and loneliness is not linear–the highest levels of loneliness were experienced in those who had been living in the UK between one and five years, which is consistent with the observations of Homoncik et al. (2017). Furthermore, the level of religious experience was significantly related to loneliness in that those with high levels of religious experience displayed lower levels of loneliness than those with low levels of religious experience. These results may suggest the need for interventions to raise awareness of potential risks among people with high levels of loneliness.

## Introduction

As many as two out of five people (41%) experienced loneliness in the last six months, and in Poland, 34% of respondents felt lonely [1]. In European countries (especially in Eastern European countries), loneliness affects up to every second person [2]. Recent estimates suggest that these problems are likely to increase by 2030 if this trend in loneliness continues. Loneliness is negatively correlated with human health but positively correlated with symptoms of depression. Understanding what may lead people to feel lonely is key in preventing and mitigating the effects of loneliness (e.g., [3, 4]).

Loneliness is defined as a discrepancy between the desired and present state of one’s social relationships [5, 6]. Many researchers have attempted to verify whether it is a homogeneous construct or whether it is possible to distinguish dimensions of loneliness [6–8]. Russel et al. [6] developed a method for examining the level of loneliness (R-UCLA) and treated it as a unidimensional phenomenon. Russel’s work had then been revised, measuring loneliness as a two-dimensional (intimate others and social network; Wilson et al. [8]) and three-dimensional (intimate others, social others, belonging and affiliation; Austin [7]) construct [9]. Through confirmatory factor analysis (CFA), the best fit indices were found for the three-dimensional model (CFI, RMSEA).

In the presented study, loneliness is understood as a discrepancy between expected and observed relationships and is understood on three levels: intimate others, social others, and belonging and affiliation (as in the Austin [7] proposal). The loneliness dimension ‘intimate others’ is understood as a feeling of rejection, severing of social ties, and experiencing unpleasant feelings of being alone. This is the most literal understanding of the term loneliness. The ‘social others’ dimension refers to the bond with loved ones (e.g., family and friends) and a feeling of deficit within the social-emotional aspect toward those people. The ‘belonging and affiliation’ dimension is related to a feeling of not being a part of a broader social group (e.g., work, neighborhood, society). The person does not feel like they have a place within a larger whole/community and are rather separate. Both internal (e.g., level of stress, level of religiosity) and external factors (e.g., labor migration, childbirth) may influence the size of the discrepancy between the expected and observed relations.

Currently, there are over 21 million Poles living abroad [10]. It is estimated that the Polish community in the UK consists of over one million Polish migrants [11]. Studies show that an increase in migration is associated with political and sociodemographic changes occurring dynamically in the world [12]. Research on the situation of Poles in the UK shows that they display lower levels of community engagement and satisfaction than those who live in Poland [11]. A lower level of social engagement abroad may directly influence feelings of loneliness, but it has not yet been verified which dimensions of loneliness are specifically sensitive to residing abroad. Both migration and loneliness are associated with a change in social context. If there is no adaptation to the new environment or circumstances, the risk of loneliness rises. Religiosity, on the other hand, is also linked to practices and social context, but it involves rituals that are not necessarily associated with a particular country or culture. An example of this is the postmigration phenomenon of the diaspora, which can serve as a connection to the country that was left behind [13]. In fact, people who formally belong to minority groups often create interactive communities close in character to those in their home country [14].

Research studies on loneliness among people living abroad have targeted several different aspects of the topic. On the one hand, predictors of loneliness, including sociodemographic data, have been studied. Those data often include the size of the locality of residence, percentage of the population accounted for by other migrants, and availability of access to cultural facilities, host country social support, media, and centers providing cultural and religious activities [15]. Among the factors directly related to the level of loneliness in migrants, time spent abroad especially should be taken into account [16]. Studies indicate that the relationship between loneliness and time spent abroad may have a curvilinear character, which in the past has often led to ambiguous results (e.g., [17–19]). It was observed that an increase in loneliness is a result of spending time abroad, but the first five years generate the strongest experiences of loneliness [20].

The second area of research on migrant loneliness is the search for risk factors of loneliness and protective factors that minimize the risks associated with feelings of loneliness. In other words, there are factors that intensify feelings of loneliness as well as those that help reduce them. An important factor that can reduce levels of loneliness is religiosity [21, 22]. People often turn to religion when they experience stressful events [23]. A coping process that takes religious or divine motives into account is called “religious coping” [24]. However, research on the effectiveness of religious coping in stressful situations often yields inconclusive results. No study published to date has attempted a quantitative synthesis of religious coping and psychological adjustment to stress [25]. The complexity of this relationship results from the fact that both religiosity and loneliness are multidimensional constructs [26]. The literature on the subject stresses the role of participation in religious community life and religious practices in reducing levels of loneliness [27]. Establishing these types of relationships helps to reduce loneliness, among other things, thanks to the received support [28]. Religious experiences, which reflect internal emotional-spiritual experiences related to the sense of God’s presence in one’s life, can also be counted as social relationships (e.g., [29]). However, no attempt has yet been made to verify whether this dimension of religiosity can also perform a function analogous to external factors (participation in the life of the community and religious practices) and thus protect against loneliness.

Religious experience is a type of internal experience in which a person has a sense of the presence or action of God. It consists of a set of sensations and experiences that form a coherent whole. It is a cognitive, aspirational, affective, and ethical process expressed in five dimensions: importance rating, entrusting in God, God’s support, openness to God, and negative experiences [30]. The importance rating indicates the relevance and significance of God in human life. Entrusting in God involves feelings of emotional connection with God and placing hope in him. God’s support denotes the positive significance of the relationship with God, especially in the face of difficult life events, when one hopes for a positive outcome in difficult matters through help received from God [31]. Openness to God refers to a person’s sensitivity to the sense of God’s presence or action. Negative experiences are understood as a sense of God’s presence when negative events cause experiences of anger, rebellion, malice, etc., to appear. This dimension does not signify the denial of God’s existence but rather feelings of negative experiences in relation to him [30].

Although it is possible to distinguish five dimensions of religious experience, two aspects, positive and negative, were analyzed in this study. The positive religious experiences consisted of importance rating, entrusting in God, God’s support, and openness to God, while the negative experiences were included in the second aspect. This division corresponds to the concept of Otto [32], who emphasizes that in religious experience, both types of feelings collide: negative (fear) and positive (fascination). Moreover, high within-group correlation and low between-group correlation showed that they do not represent opposite poles [30].

### Problem

The present study aims to verify differences in the level of loneliness at three key stages of labor migration: up to one year, between one and five years (when one realizes that the job is not just temporary and that it generates the need to stay outside of the home country, as it is more difficult to return to it as anything other than a guest), and more than five years (when a person accepts that working abroad is part of their life). The key periods of labor migration were inspired by the stages of migration proposed by Homoncik et al. [20]. The time up to one year after moving is the period when a person decides to look for employment outside of the country, most often treating it as a transitional and temporary issue. At the same time, it is a trial period in which deficits in social-emotional relations, as well as plans to return to the homeland, family, and loved ones, may not yet appear. The period of one to five years is a time of potential crises related to conflicts of professional and social roles and family issues affecting the formation of social relationships. At the same time, it may be a period of social identity crisis and self-questioning (*Who am I*? *What is my place*? *Am I happy*?). An individual often evaluates working abroad in an emotional way, forming a process which could be additionally burdened by stress, a sense of longing for family, and loneliness. The next and final stage associated with staying abroad is acceptance of the living situation and adaptation that comes with it.

The present study verifies whether the time of crisis (one to five years abroad) coincides with the highest level of perceived loneliness compared to periods up to one year and over five years. (H1). The role of religious experience in feeling loneliness among people residing abroad is also raised. In this context, it is conjectured that people with high levels of positive religious experience are characterized by lower levels of loneliness compared to those with low levels of positive experience (H2). Conversely, those with low levels of negative religious experiences are characterized by lower levels of loneliness compared to those with high levels of negative experiences (H3). Since religious experience is a phenomenon that changes over time, the interaction of religious experience with time spent abroad in explaining migrants’ sense of loneliness in the UK is verified (H4). Religious experience and time spent abroad may both exacerbate the level of loneliness and reduce that level; therefore, verification of this hypothesis seems particularly important.

## Materials and methods

### Participants

The participants in this study were native Poles (N = 200; 105 men and 95 women). The sample size was determined by the G*Power program (v. 3.1.9.7, see Appendix B in S1 Appendix for protocol of required sample size analysis). Participants were aged between 40 and 59 years (M = 48.69; SD = 5.35). For men, the mean age was M = 50.03; SD = 5.35, while for women, the mean was M = 47.20; SD = 4.97. There were no missing data in the study–all questionnaires were fully completed. Participants were separated into three groups on the basis of time spent abroad: up to one year (N = 49), between one and five years (N = 79) and more than five years (N = 72). Taking into account the place of residence of the participants, 39.5% live in rural areas, 37% live in small cities (up to 150,000 residents), and 23.5% of respondents are from the largest urban centers (more than 150,000 residents). Half of the respondents declare their knowledge of English to be at the intermediate level, 30.5% of respondents describe their level of proficiency in the language as advanced, and 19.5% of respondents say they have no knowledge of the language or only know single words. Just over half of the respondents (52%) are married, 22% of the respondents are single, and 16.5% of the sample are divorced. The least numerous group is widowers (9.5%). Almost half of the respondents live abroad with their immediate family (48.5%), while 28% of respondents live with relatives/friends or acquaintances. Those living alone account for 23.5% of the sample. Just over half of the respondents (52.5%) belong to a religious community, 52% of respondents describe themselves as both believers and practitioners, while 48% describe themselves as only believers and nonpractitioners. The majority of respondents (56.5%) say they have free access to Catholic Church abroad. On the other hand, 32% of respondents admit that this access is difficult, e.g., due to distance from home or working hours. Lack of access to Catholic Church was declared by 11.5% of participants.

Quantitative research was carried out using a distributed survey method and purposive sampling (January-March 2022). Respondents provided their answers to the test items included in the paper questionnaire and completed it by hand. They gave verbal consent to take part in the study and were assured of its anonymity, confidentiality, and scientific nature. The Committee for Research Ethics of the Institute of Psychology at the John Paul II Catholic University of Lublin gave their approval for the proposal of the presented research prior to its start (ref. no.: KEBN 27/2022).

### Tools

#### Revised UCLA loneliness scale (R-UCLA)

The R-UCLA, which was used to assess loneliness, was proposed by Kwiatkowska et al. [9] based on the model of Russel et al. [6]. It proved to have high content validity, as well as an adequate level of reliability and external validity. The R-UCLA examines loneliness expressed in three dimensions: intimate others (10 items, Cronbach’s alpha = .84), social others (5 items, Cronbach’s alpha = .80), and belonging and affiliation (5 items, Cronbach’s alpha = .65) on a four-point scale (1 = “never” 2 = “rarely” 3 = “sometimes” 4 = “often”) (for test items see Appendix in Kwiatkowska et al. [9], pp. 170).

#### Religious experience scale (RES)

Religious experience is a holistic event containing components of cognition, orientation, information, emotions, motivation, decisions, actions and expression. It refers to individual and direct perception of the supernatural reality. Combined with conviction about the certainty and truthfulness of what happens during intentional and positive relationships with God, religious experience is, however, limited by elements of mystery. The RES, proposed by Tatala and Walesa [30], is one of the methods for measuring religious experience, and it has a high level of content validity. It comprises five scales: importance rating (4 items, Cronbach’s alpha = .84), negative experiences (4 items, Cronbach’s alpha = .77), God’s support (3 items, Cronbach’s alpha = .84), entrusting in God (3 items, Cronbach’s alpha = .83) and openness to God (3 items, Cronbach’s alpha = .69). The responses are given on a five-point scale: 1 = “strongly disagree”, 2 = “disagree”, 3 = “sometimes agree, sometimes disagree”, 4 = “agree”, and 5 = “strongly agree” (for test items, see Appendix A in S1 Appendix in this paper).

## Results

In preliminary analyses, correlations between the religious experience scales and loneliness scales were verified (Table 1).

**Table 1:** Pearson correlations among religious experience scales and loneliness scales.

	Intimate others	Social others	Belonging and affiliation	Importance rating	Negative experiences	God’s support	Entrusting in God	Openness to God
Intimate others	1	.34**	.37**	-.03	.26**	-.06	-.03	.09
Social others		1	.63**	-.23**	.11	-.26**	-.33**	-.29**
Belonging and affiliation			1	-.16*	.12	-.16*	-.24**	-.17*
Importance rating				1	.03	.81**	.79**	.72**
Negative experiences					1	-.002	.04	.13
God’s support						1	.81**	.75**
Entrusting in God							1	.76**

Note: ** p < .01

Because the positive religious experience scale group is highly correlated, the subsequent analyses were conducted using the overall positive experience score. It comprises the average of the four experience scales: entrusting in God, God’s support, openness to God, and the importance rating. The negative experiences scale, on the other hand, is formed by a single scale containing 4 test items. In the next step, the mean was calculated for the two newly emerged scales (positive experiences and negative experiences), based on which a distinction was made between those with low and high levels of positive experiences (M = 41.47; SD = 12.22), as well as low and high levels of negative experiences (M = 9.92; SD = 3.38). The main part of the analyses examined the differences in perceived loneliness due to time abroad (H1), level of positive religious experiences (H2), level of negative religious experiences (H3), and interaction between time abroad and level of positive and negative religious experiences (H4).

The statistical model used for the analyses was 3-factor MANOVA, and it involved three between-subject variables: time abroad, level of positive experiences and level of negative experiences. The dependent variables were the subscales of the loneliness scale: intimate others, social others, and belonging and affiliation. The following section discusses the results for each of the aforementioned dependent variables by analyzing both main effects and interactions to verify hypotheses H1-H4. For the dependent variable ‘intimate others’, the main effect of negative religious experiences was observed (F(1, 188) = 8.77; p < .01; η^2^ = .05). Those with high levels of negative religious experiences (M = 2.33; SD = 0.54) displayed higher levels of intimate others compared to those with low levels of negative experiences (M = 2.04; SD = .58). There was no effect of positive religious experiences or time abroad. The interaction between time abroad (T) * level of negative experiences (N) * level of positive experiences (P) was found to be significant (F(2, 188) = 4.09; p < .05; η^2^ = .04). A pairwise comparison analysis with Bonferroni correction was conducted to verify which comparisons were significantly different from each other (Table 2).

**Table 2:** Post hoc comparisons–Time abroad (T) * level of Negative experiences (N) * level of Positive experiences (P).

Condition	Comparison between the variables	*p* <
N↓P↓	T1 (M = 1.83)–T2 (M = 2.24)	.100
N↑P↑	T1 (M = 1.98)–T2 (M = 2.52)	.100
T1 P↓	N↓ (M = 1.83)–N↑ (M = 2.23)	.050
T2 P↑	N↓ (M = 1.99)–N↑ (M = 2.52)	.010
T3 P↑	N↓ (M = 1.96)–N↑ (M = 2.42)	.050
T1 N↓	P↓ (M = 1.83)–P↑ (M = 2.25)	.100

A detailed analysis showed that interaction occurred only in three circumstances. Those with high levels of negative experiences displayed higher levels of intimate others compared to those with low levels of negative experiences when (1) they were abroad for up to a year (T1) and were characterized by an overall low level of positive religious experiences (P↓), (2) they had been abroad for one to five years (T2) and had a high level of positive religious experience (P↑), and (3) they had been abroad for more than five years (T3) and had a high level of positive religious experience (P↑).

The variable ‘social others’ was significantly (or at the level of statistical tendency) differentiated by time (T) (F(2, 188) = 2.44; p < .1; η^2^ = .03), negative religious experience (N) (F(1, 188) = 3.65; p < .1; η^2^ = .02), and positive religious experience (P) (F(1, 188) = 10.81; p < .01; η^2^ = .05). Pairwise comparison showed that time abroad differentiated the results for social others (p < .1): those who had been abroad between one and five years were characterized by higher levels of social others (M = 2.09; SD = .70) compared to those who had been abroad longer (M = 1.89; SD = .58). Those with low levels of negative religious experiences were characterized by lower levels of social others (M = 1.90; SD = .66) compared to those with high levels of negative religious experiences (M = 2.10; SD = .64) (p < .1). Individuals with low levels of positive religious experiences were characterized by higher levels of social others (M = 2.14; SD = .74) compared to individuals with high levels of positive religious experiences (M = 1.84; SD = .51) (p < .01).

The variable ‘belonging and affiliation’ was significantly (or at the level of statistical tendency) differentiated by time (T) (F(2, 188) = 3.53; p < .05; η^2^ = .04), negative religious experience (N) (F(1, 188) = 3.24; p < .1; η^2^ = .02), and positive religious experience (P) (F(1, 188) = 6.78; p < .05; η^2^ = .04).

Pairwise comparisons revealed that time abroad differentiated the results for belonging and affiliation (p < .1), such that those staying abroad for one to five years were characterized by higher levels of belonging and affiliation (M = 2.25; SD = .59) compared to those who had been abroad longer (M = 2.05; SD = .48). Those with low levels of negative religious experiences were characterized by lower levels of belonging and affiliation (M = 2.05; SD = .57) compared to those with high levels of negative religious experiences (M = 2.22; SD = .56) (p < .1). Those with low levels of positive religious experiences were characterized by higher levels of belonging and affiliation (M = 2.23; SD = .61) compared to those with high levels of positive religious experiences (M = 2.02; SD = .52) (p < .05).

## Discussion

A growing number of people are being affected by loneliness. According to the literature, loneliness has a significant impact on several physical and mental health issues, such as depression, cardiovascular problems and general health [33, 34]. Loneliness is estimated to cost UK employers £2.5 billion each year [35]. People are experiencing loneliness at an increasingly younger age, which contradicts the stereotype of associating loneliness with late old age when its symptoms intensify due to fear of illness, death, and passing of loved ones. A number of factors can be attributed to this phenomenon, including socioeconomic changes taking place in the world. The global economic situation has caused many people to migrate for labor to improve their quality of life. In addition to the undoubted benefits, the decision to leave the country of origin brings risks, such as loneliness caused by separation from loved ones and the community. Loneliness hinders proper functioning in various areas: social, emotional, and cognitive [36, 37]. This became the reason for undertaking the presented research on protective factors against loneliness experienced by Polish migrants in the UK. Religiosity is understood as a buffer that plays this protective role in a direct, intimate relationship with God and while participating in worship and religious practices. Thus, religious experience was considered to be a particularly important factor in the study (e.g., [38]).

The goal of this study was to examine the interaction between time abroad and religious experience and their joint effect on loneliness, which was analyzed in three dimensions: intimate others, social others, and belonging and affiliation [7]. The interaction effect was observed only for intimate others. It was found that negative experiences increased people’s feelings of loneliness. ‘Social others’ was significantly (or at the level of statistical tendency) differentiated by time, negative religious experience, and positive religious experience. Pairwise comparisons showed that those who had stayed abroad between one and five years had higher levels of social others compared to those who had been abroad longer [25]. Individuals with low levels of negative religious experiences showed lower levels of social others. Those with low levels of positive religious experiences were characterized by higher levels of social others. The ‘belonging and affiliation’ variable was significantly (or at the level of statistical tendency) differentiated by time, negative religious experience, and positive religious experience. Length of time abroad differentiated the results for belonging and affiliation such that those who had stayed abroad between one and five years had higher levels of this variable. Those with low levels of negative religious experiences were characterized by lower levels of belonging and affiliation. Those with low levels of positive religious experiences were characterized by higher levels of belonging and affiliation (e.g., [39]).

The presented findings are consistent with previous observations on the potentially protective role of religiosity against experiencing loneliness [40, 41]. However, the results of other studies examining the mediating effect of religiosity on the relationship between loneliness and time abroad are inconclusive. For example, Ciobanu and Fokkema [40] concluded that the importance of religiosity did not vary at different stages of being abroad. Homoncik et al. [20], on the other hand, indicated that the level of loneliness of people living abroad is dependent on the length of stay: the highest level of loneliness was reported between the first and fifth years after leaving the home country. Thus, especially for this period of residing abroad, the role of protective factors against loneliness, including religiosity, may be important. Religious styles identified by Gallagher and Trzebiatowska [42] may partly explain the variation in coping with loneliness. Some people cultivate their cultural habits after going abroad, some use their time away to explore faith, and others abandon religion, thus losing one of the potential forms of support (e.g., [43]). The results revealed a nonlinear pattern of loneliness in relation to time abroad. The period of particularly high levels of loneliness experienced is one to five years. This period is followed by adaptation to the living situation [20]. It was confirmed that religiosity, especially religious experience, is a protective factor and a buffer against feeling lonely. This observation is consistent with the literature, according to which religiosity allows for an easier passage through crises, difficulties (e.g., [29, 30]), or labor migration. To cope with stressful situations, a person may engage in religion-based activities such as worshipping, praying, or secular activities such as playing sports or traveling. This notion was confirmed by a number of studies: a positive relationship between religiosity and psychological well-being was observed in a sample of Muslims from Pakistan [40]. At the same time, a strong negative relationship between religiosity and loneliness and between religiosity and anxiety was found. In another study, positive and negative forms of religious coping were related to positive and negative psychological adjustment to stress, respectively [25]. As people living abroad often experience a sense of exclusion, religion and religious practices may serve as coping strategies. This is supported by the research of Aydin et al. [39], who found that people who had been socially isolated often turned to religion to cope with their situation.

Due to the novel character of the presented study, the results should be interpreted with caution. First, the length of stay abroad was not expressed as a continuous variable but as three key stages: up to one year, one to five years, and more than five years. This division is based on observations of Homoncik et al. [20], who determined that these three stages are key moments of migration. Nonetheless, it would be worthwhile to design a longitudinal study to verify the impact of time abroad on loneliness. Second, the sample of participants was a group of Polish migrants in the UK, but there is evidence that levels of loneliness can vary on the basis of the type of culture–individualistic or collectivist. It would therefore be valuable to conduct a study with migrants of different cultures. Third, the reasons behind the decision to go abroad also seem to be particularly important, as there is evidence that people who were forced to leave their home country experience greater levels of loneliness than those who were not [44]. Previous levels of loneliness and religious experience from when respondents resided in their country of origin were not determined and therefore could not be controlled. Without this information acting as a baseline, it is difficult to estimate the actual contribution of the two variables, migration and religious experience, in explaining loneliness. Finally, another aspect that may moderate the level of perceived loneliness is the personality traits of the subjects, as these are closely related to experiencing loneliness [45] and religiosity [27].

In the future, it would be worthwhile to verify how diaspora is related to feelings of loneliness associated with migration in the context of the findings presented in this paper. In particular, it may be of interest whether religious experience is significantly related to strong ties to one’s homeland and the ‘origin’ characteristic of diasporas. Both of these issues are strongly connected due to cultural backgrounds and therefore may be characterized by interaction effects on migrants’ levels of loneliness.

The present study verified the relationship between loneliness and religiosity, including the effect of time abroad. It was possible to show that the relationship between time abroad and loneliness is curvilinear. Moreover, religious experience was found to be important in reducing loneliness at every stage of living abroad, even for the highest loneliness risk period, between one and five years of being abroad.

## Supporting information

S1 File (SAV)

S1 Appendix (DOCX)
