# Supplementary material for: Loneliness and time abroad in Polish migrants in the UK: Protective role of religious experience
Source: PLoS One. 2023 Feb 15;18(2):e0279984. doi: 10.1371/journal.pone.0279984 (PMC9931139; doi:10.1371/journal.pone.0279984)
Supplement: S1 Appendix — (DOCX) [file pone.0279984.s002.docx]

Appendices

Appendix A

Religious Experience Scale (RES) (Małgorzata Tatala & Czesław Walesa)

The statements below concern religious experiences. Read them and mark to what extent the statement applies to you by circling around the digit that indicates your answer. There is a five-point scale. There are no good or bad answers, and every answer counts as long as it is honest and expresses your personal beliefs. The research is completely anonymous, and the results will be used for scientific purposes only.

1 = “strongly disagree”
2 = “disagree”
3 = “sometimes agree, sometimes disagree”
4 = “agree”
5 = “strongly agree”

1. I feel that suffering makes sense to me if I refer it to Jesus Christ/Doświadczam tego, że cierpienie ma dla mnie sens, wtedy gdy odnoszę je do Jezusa Chrystusa
2. Despite any indifference I may experience, I remain with God/Mimo przeżywanego zobojętnienia trwam przy Bogu
3. Difficult life situations make me deepen my relationship with God/Trudne sytuacje życiowe skłaniają mnie do pogłębienia więzi z Bogiem
4. I pass important religious messages on to others/Przekazuję innym ważne treści religijne
5. I experience God overcoming all of my limitations/Doświadczam tego, że Bóg pokonał wszystkie moje ograniczenia.
6. In hopeless situations, I see the intervention of God/W sytuacjach beznadziejnych dostrzegam interwencję Pana Boga.
7. Life only makes sense to me with God/Życie ma dla mnie sens jedynie w Bogu
8. In difficult situations, I see the presence of God/W sytuacjach trudnych dostrzegam obecność Boga.
9. I want to have more confidence in my faith/Pragnę mieć więcej pewności w wierze
10. In life’s adversities, I abide by my faith in God/W przeciwnościach życiowych podtrzymuję moją wiarę w Boga
11. When I experience a great misfortune, my relationship with God breaks down/Gdy przeżywam wielkie nieszczęście, moja więź z Bogiem ulega załamaniu
12. I experience that God comes to me in silence/Doświadczam tego, że Bóg przychodzi do mnie w ciszy
13. I often ask of myself the ultimate purpose to my life/Często zadaję sobie pytanie o ostateczny cel mojego życia
14. Fulfilment of my vocation is my personal call from Jesus Christ/Realizowanie mojego powołania jest dla mnie wezwaniem Jezusa Chrystusa
15. In the most difficult moments of my life, I do not experience God’s intervention/W najtrudniejszych chwilach życia nie doświadczam interwencji Boga
16. In situations of suffering, I experience defiance against God/W sytuacjach cierpienia przeżywam bunt wobec Boga/
17. I feel anger toward God because of the deaths of my close ones/Przeżywam złość wobec Boga z powodu śmierci bliskich mi osób

Scoring key for the subscales:

Importance rating/Ocena ważności: 01, 04, 07, 14

Negative experiences/Przeżycia negatywne: 11, 15, 16, 17

God’s support/Wsparcie od Boga: 05, 06, 08

Entrusting in God/Zaufanie Bogu: 02, 03, 10

Openness to God/Otwartość na Boga: 09, 12, 13

Appendix B

**Analysis: A priori: Compute required sample size**

**Input:**

Effect size f = 0.40

α err prob = 0.05

Power (1-β err prob) = 0.95

Number of groups = 12

**Output:**

Noncentrality parameter λ = 26.88

Critical F = 1.85

Numerator df = 11

Denominator df = 156

Total sample size = 168

Actual power = 0.95
